# In Vivo and Ex Vivo Mitochondrial Function in COVID-19 Patients on the Intensive Care Unit

**DOI:** 10.3390/biomedicines10071746

**Published:** 2022-07-20

**Authors:** Lucia W. J. M. Streng, Calvin J. de Wijs, Nicolaas J. H. Raat, Patricia A. C. Specht, Dimitri Sneiders, Mariëlle van der Kaaij, Henrik Endeman, Egbert G. Mik, Floor A. Harms

**Affiliations:** 1 Laboratory of Experimental Anesthesiology, Department of Anesthesiology, Erasmus MC, University Medical Center Rotterdam, 3000 CA Rotterdam, The Netherlands; c.dewijs@erasmusmc.nl (C.J.d.W.); n.raat@erasmusmc.nl (N.J.H.R.); p.specht@erasmusmc.nl (P.A.C.S.); d.sneiders@erasmusmc.nl (D.S.); m.vanderkaaij@erasmusmc.nl (M.v.d.K.); e.mik@erasmusmc.nl (E.G.M.); f.harms@erasmusmc.nl (F.A.H.); 2 Department of Intensive Care, Erasmus MC, University Medical Center Rotterdam, 3015 GD Rotterdam, The Netherlands; h.endeman@erasmusmc.nl

**Keywords:** mitochondrial function, mitochondrial oxygen tension, mitochondrial oxygen consumption, mitochondrial respiration, mitochondrial DNA, COVID-19

## Abstract

Mitochondrial dysfunction has been linked to disease progression in COVID-19 patients. This observational pilot study aimed to assess mitochondrial function in COVID-19 patients at intensive care unit (ICU) admission (T1), seven days thereafter (T2), and in healthy controls and a general anesthesia group. Measurements consisted of in vivo mitochondrial oxygenation and oxygen consumption, in vitro assessment of mitochondrial respiration in platelet-rich plasma (PRP) and peripheral blood mononuclear cells (PBMCs), and the ex vivo quantity of circulating cell-free mitochondrial DNA (mtDNA). The median mitoVO_2_ of COVID-19 patients on T1 and T2 was similar and tended to be lower than the mitoVO_2_ in the healthy controls, whilst the mitoVO_2_ in the general anesthesia group was significantly lower than that of all other groups. Basal platelet (PLT) respiration did not differ substantially between the measurements. PBMC basal respiration was increased by approximately 80% in the T1 group when contrasted to T2 and the healthy controls. Cell-free mtDNA was eight times higher in the COVID-T1 samples when compared to the healthy controls samples. In the COVID-T2 samples, mtDNA was twofold lower when compared to the COVID-T1 samples. mtDNA levels were increased in COVID-19 patients but were not associated with decreased mitochondrial O_2_ consumption in vivo in the skin, and ex vivo in PLT or PBMC. This suggests the presence of increased metabolism and mitochondrial damage.

## 1. Introduction

The severe acute respiratory syndrome coronavirus 2 (SARS-CoV-2) first emerged in Wuhan, China. This single-stranded RNA coronavirus has caused the coronavirus disease 2019 (COVID-19), resulting in a pandemic which spread widely and has infected over 436 million individuals and resulted in almost 6.4 million deaths [1]. COVID-19 is characterized by the development of a respiratory tract infection and, in severe cases, a viral sepsis, caused by a surge in inflammation known as a cytokine storm [2]. A major hallmark of severe COVID-19 patients is the disturbed tissue oxygenation which is a direct result of this life-threatening immune response towards SARS-CoV-2. This phenomenon will, without treatment, ultimately result in (multi) organ dysfunction [3].

Saleh et al. found that the cytokine storm of COVID-19 sepsis results in mitochondrial dysfunction due to an increase in mitochondrial reactive oxygen species (ROS) generation. These ROS directly stimulate the production of proinflammatory cytokines [2]. These proinflammatory cytokines drive oxidative stress and ROS generation, leading to a vicious inflammatory and oxidation cycle in which damaged mitochondria cause further mitochondrial injury [2,4]. These findings suggest that mitochondrial dysfunction may play a role in the pathophysiology of COVID-19. A similar phenomenon of impaired cellular/mitochondrial metabolism with adequate oxygenation was coined as “cytopathic hypoxia” by Fink et al. in the late 1990s [5].

In order to substantiate the different hypotheses of mitochondrial dysfunction in COVID-19 disease, insight into in vivo mitochondrial function is essential. The novel Cellular Oxygen METabolism (COMET) monitor enables noninvasive, in vivo measurement of the mitochondrial oxygen tension (mitoPO_2_) and mitochondrial oxygen consumption (mitoVO_2_). The measuring method is based on the natural delayed fluorescence of protoporphyrin IX (PpIX) [6] and has been extensively validated in animal models and for human use [7,8,9,10,11]. The mitoPO_2_ reflects the local balance between oxygen supply and consumption [12]. The mitoVO_2_ is a measure of mitochondrial oxygen consumption by means of measuring oxygen disappearance rate. This disappearance rate provides an indication of aerobic mitochondrial function [12].

The aim of this study is to analyze whether in vivo mitochondrial function is altered in COVID-19 patients. To this end, COMET measurements conducted in COVID-19 patients will be compared to age- and gender-matched healthy volunteers and patients without COVID-19 undergoing general anesthesia. The mitoVO_2_ in the general anesthesia group was measured after induction of anesthesia prior to the start of cardiothoracic surgery.

Additionally, the COMET measurements of COVID-19 patients and healthy volunteers will be compared with ex vivo mitochondrial function measurements [13]. These ex vivo mitochondrial function measurements were conducted by using high-resolution respirometry (Oxygraph O2k) to measure mitochondrial oxygen consumption in platelet-rich plasma (PRP) and peripheral blood mononuclear cells (PBMCs). Previous studies utilizing the same technique examined the basal and maximal mitochondrial respiration of PBMCs from COVID-19 patients and demonstrated that COVID-19 patients had a reduced basal and maximal respiration, reduced proton leak, and reduced spare capacity in monocytes [14,15]. These findings imply that COVID-19 resulted in dysfunctional and metabolically impaired mitochondria.

Furthermore, utilizing free mitochondrial DNA (mtDNA) in plasma as a biomarker for disease severity in sepsis has been a subject of interest in various studies [16,17,18]. This implies that mtDNA can potentially be a useful biomarker in COVID-19 [19]. The potential association between mtDNA and SARS-CoV-2 infection is attributed to the function of mtDNA as a damage-associated molecular pattern (DAMP). It is believed that SARS-CoV-2 replicates in the mitochondria, causing membrane permeability and leak of mtDNA [20]. In turn, the leak of mtDNA provokes local and systemic inflammation [20]. To further investigate this correlation the mtDNA in COVID-19 patients and healthy volunteers will be analyzed.

To summarize, the primary aim of this study is to compare COMET measurements of in vivo mitochondrial function in ICU-admitted COVID-19 patients versus those of a matched group of healthy volunteers and a general anesthesia cohort. Furthermore, in vivo mitoVO_2_ measurements will be compared to ex vivo measurements of mitochondrial respiration in platelet-rich plasma (PRP) and peripheral blood mononuclear cells (PBMCs), and mtDNA in plasma from COVID-19 patients will be compared to the levels of matched healthy volunteers.

## 2. Material and Methods

### 2.1. Study Design and Setting

The noninvasive measurement of mitochondrial function in vivo (NIMFO) in septic patients (registered in the Netherlands Trial register under NL9631) is a single-center, prospective, observational trial. It was approved by the Medical Ethics Committee of the Erasmus Medical Centre (MEC 2016-540, NL58587.078.16). All study procedures were performed in accordance with the relevant guidelines and regulations.

The inclusion period of the COVID-19 patients and healthy age-matched volunteers started in January 2021 and went until July 2021. The trial was performed in the Erasmus Medical Center (EMC), a tertiary university medical center situated in the Netherlands.

Informed consent from patients and legal representatives was acquired using a deferred proxy consent construction. All healthy volunteers, legal representatives, and patients signed the informed consent form, unless patients passed away before informed consent could be sought.

For the reason that the Erasmus Medical Center is a tertiary hospital, the stage of disease in which the patient was included could not be standardized. However, patients could only be included within 72 h of ICU admission with COVID-19. Furthermore, the patients needed to be over 18 years old and younger than 90 years of age. Healthy controls were matched in age (±5 years) and gender to the included COVID-19 patients and were only included if they did not have any relevant comorbidities (maximum class 2 of the American Society of Anesthesiologists (ASA) classification). Exclusion criteria for both groups included porphyria and presence of mitochondrial disease. In addition to this, healthy volunteers were excluded if there was the presence of COVID-19-related symptoms, a positive COVID-19 polymerase chain reaction (PCR) test less than one month prior, or if they had received a COVID-19 vaccination less than two weeks prior. These COVID-19-related exclusion criteria were chosen in order to reduce the chance of possible confounders.

This manuscript also contains unpublished mitoPO_2_ and mitoVO_2_ data from another study in patients undergoing cardiothoracic surgery. This study was approved by the Medical Ethics Committee of the Erasmus Medical Centre (MEC 2017-532, NL62551.078.17). All study procedures were performed in accordance with the relevant guidelines and regulations. A total of 9 out of the 41 patients which were included in the aforementioned study were used in this manuscript. This is due to the fact that the mitoVO_2_ in this subgroup of patients was measured after the induction of anesthesia and before the start of surgery. By including this cohort in this manuscript, the effects of anesthetic agents during general anesthesia can be illustrated. For the purposes of this paper, this patient cohort will be referred to as the “general anesthesia group”. The methods by which the mitoVO_2_ and mitoPO_2_ were measured were identical (description further on in the methods section) except for the location of the measurement. Due to logistical constraints, given the fact that the patients receive a sternotomy for their cardiothoracic surgical procedure, the COMET measurements were conducted on the upper arm instead of the sternum. The inclusion criteria for the aforementioned study were over 18 years age, acceptable proficiency in the Dutch language, and cardiac surgery requiring a cardiopulmonary bypass. The exclusion criteria were pregnancy or lactation, skin lesions on the upper arm or shoulder that could impede the mitoPO_2_ measurements, not having an indication for invasive intra-arterial blood pressure monitoring, emergency surgery, intracardiac shunts, or the presence of mitochondrial disease. Lastly, patients eligible and willing to participate signed informed consent forms prior to their surgery.

### 2.2. Variables

Included patients were measured at two time points. The first measurement (T1) was performed within 72 h after admission to the ICU of the Erasmus MC, preferably within 24 h. The second measurement (T2) was performed seven days after the first. The healthy controls were measured once. Patient screening was performed by the research team using the electronic medical dossier. The Sequential Organ Failure Assessment (SOFA) score was scored at the date and time of ICU admission to the ICU of the Erasmus Medical Center [21]. Similar to the SOFA score, the category of acute respiratory distress syndrome (ARDS) was scored at admission to the ICU of the Erasmus Medical Center and based upon the Berlin definition [22]. The Acute Physiology and Chronic Health Evaluation (APACHE) II score was scored at T1 and T2 [23].

Oxygen measurements were performed by means of the COMET*^®^* monitor (Photonics Healthcare, Utrecht, The Netherlands). First, a self-adhesive patch containing 8 mg 5-aminolevulinic acid (ALA) (Alacare, Photonamic GmbH und Co. KG, Pinneberg, Germany) was applied on the skin of the sternum. The ALA causes an upregulation of PpIX, enabling the measurements with the COMET*^®^* monitor. To enhance ALA penetration, adequate skin preparation proved essential. Hair was shaved (if present) and the skin was rubbed with a fine abrasive pad of a standard ECG sticker to remove the top parts of the stratum corneum. ALA was applied for at least five hours to enable a suitable concentration of PpIX to be synthesized. During the application time, direct sunlight exposure was avoided.

In addition to measuring mitoVO_2_, a blood sample (K2EDTA 10 mL tubes (BD Vacutainer*^®^*, Becton Dickinson, Plymouth, UK)) was collected for the measurement of mitochondrial function in platelets, peripheral blood mononuclear cells, and mitochondrial DNA in plasma as a marker of mitochondrial damage.

### 2.3. MitoPO_2_ and mitoVO_2_ Measurements

The methodology behind the mitoPO_2_ measurements have previously been described by F.A. Harms et al. “The background and principles of the PpIX-TSLT are described in detail elsewhere [6,8,24]. In short, PpIX is the final precursor of heme in the heme biosynthetic pathway. PpIX is synthesized in the mitochondria, and administration of 5-aminolevulinic acid (ALA) enhances the PpIX concentration. Since the conversion of PpIX to heme is a rate-limiting step, administration of ALA causes accumulation of PpIX inside the mitochondria. PpIX possesses a triplet state that reacts strongly with oxygen, making its lifetime oxygen-dependent. Population of the first excited triplet state occurs upon photo-excitation with a pulse of light and causes the emission of red delayed fluorescence. The delayed fluorescence lifetime is related to mitoPO_2_ according to the Stern–Volmer equation:

$${\mathrm{PO}}_{2}=\frac{\frac{1}{\tau }-\frac{1}{\tau_{0}}}{k_{q}}$$

in which *τ* is the measured delayed fluorescence lifetime, *k_q_* is the quenching constant, and *τ*_0_ is the lifetime at zero oxygen” [12]. The Stern–Volmer equation is valid for a homogenous oxygen distribution and after excitation with a pulse of light of which the lifetime is much shorter than *τ.* F.A. Harms goes on to further describe that “in case of a non-homogenous oxygen distribution inside the measurement volume, a reliable estimation of the average PO_2_ can be made by the rectangular distribution method (RDM)’’ [12,25,26].

MitoVO_2_ measurements were performed by local occlusion of the microcirculation in the tissue. This was achieved by applying localized pressure with the measuring probe of the COMET on the measurement site. “This simple procedure created stopped-flow conditions and induced measurable oxygen disappearance due to cessation of microvascular oxygen supply and ongoing cellular oxygen consumption. MitoPO_2_ was measured, before and during application of pressure” [27], by repeated measurements at a rate of 2 Hz using a single laser pulse per individual mitoPO_2_ measurement. The rate of mitoPO_2_ change during stopped-flow conditions was determined from the linear part of the curve directly after the beginning of tissue compression. MitoVO_2_ was calculated as ∆ mitoPO_2_/∆t [9].

### 2.4. Sample Preparation

A whole blood count (WBC) was conducted with a Beckman Coulter DxH500 (Beckman Coulter, Brea, CA, USA). Plated-rich plasma (PRP) and peripheral blood mononuclear cells (PBMCs) were isolated from the same blood tube and isolation started within 1, 2 h after the blood samples were taken. Our analysis in platelets was based on previously published methods for measurement of mitochondrial respiration in platelets [28,29,30]. Compared to the described protocols we did see the benefit of first creating a platelet pellet and resuspending it in the same plasma. Therefore, we altered the platelet isolation protocol used in these studies, creating a PRP instead of a near protein-free plasma and platelet pellet. The method used for isolation of PBMC was based on the MiPNet 21.17 blood cell isolation protocol for high-resolution respirometry (HRR) with slight modifications, as described below [31]. All procedures were performed at room temperature.

After centrifuging at 150× *g* for 15 min (acceleration 9, brake 9), 1.1 mL of the top layer of the supernatant was removed for collecting PRP. The residue (hematocrit plus PRP) was centrifuged for 6 min at 4000× *g* (acceleration 9, brake 9) and all plasma was collected. For the determination of free mitochondrial DNA in plasma as a marker of mitochondrial damage [32], 250 µL of this plasma was divided over two 0.5 mL sterile tubes, immediately snap frozen in liquid nitrogen, and stored at −80 ºC for measurements at a later time.

After isolation of PRP, the residue of blood was gently mixed for isolation of PBMCs and divided over two Leucosep™ tubes 12 mL (Greiner Bio-one, Frickenhausen, Germany) using Lymphoprep ™ (Biovision, Milpitas, CA, USA) as separation medium. Blood was diluted 1:1 with phosphate-buffered saline, mixed gently (DPBS Dulbecco, Biowest, Nuaillé, France)), and centrifuged at 800× *g* for 20 min (acceleration 6, brake 1). Then, the buffy coat was collected with a Pasteur pipette and the isolated PBMCs were washed twice in 14 mL DPBS and centrifuged at 250× *g* for 10 min (acceleration 6, brake 1). After the last washing step, the pellet was resuspended in 2.4 mL RPMI-1640 medium from Merck (Darmstad, Germany) or Gibco (Paisley, UK). The isolated PBMC concentration was calculated by combining the measured lymphocyte and monocyte counts from the Beckman Coulter DxH500. When necessary, the PBMC cell suspensions were diluted with RPMI-1640 medium to obtain a concentration of 2.0–2.5 × 106/mL.

### 2.5. High-Resolution Respirometry

Directly after isolation, mitochondrial oxygen consumptions rates (OCR) of intact platelets and PBMCs were measured using a high-resolution respirometer (Oxygraph O2k: Oroboros Instruments, Innsbruck, Austria). Prior to cell suspension loading, a volume calibration was performed and the instrument was calibrated following manufacturer instructions with 1.1 mL plasma (platelets) or 1.5 mL RPMI-1640 medium (PBMCs). An oxygen solubility factor of 0.89 was used to calculate oxygen levels in plasma and RPMI-1640 medium. After calibration, 1 mL of PRP was added to the chamber. For the PBMCs, the RPMI-1640 medium was removed from the chamber and filled with 2.1 mL PBMC suspension. The chamber was closed and equilibrated, and a coupling-control protocol was applied to study mitochondrial function. The definitions of the various mitochondrial respiration states are described by Gnaiger et al. [33]. ROUTINE respirations of unstimulated platelets in plasma and PBMCs in RPMI were estimated at 37 °C with a stirring speed of 750 rpm. All experiments were performed at O2 concentrations >50 µM to avoid oxygen-dependent respiration [34,35]. When the oxygen level dropped below 50 µM, the chamber stopper was raised for reoxygenation. 

All chemicals for the mitochondrial experiments were purchased from Sigma-Aldrich (St. Louis, MO, USA). Oligomycin was added to the chamber (5 mM stock; 2 µL for platelets and 1 µL for PBMCs), effectively blocking ATP synthase activity to measure non-ATP-linked (LEAK) respiration. Subsequently, serial additions of the uncoupler carbonyl cyanide p-trifluoromethoxy phenylhydrazone (FCCP) (20 mM stock in steps of 1 µL for platelets and 1 mM stock in steps of 0.5 µL for PBMCs) were added until a maximal (MAX) respiration rate was obtained. FCCP additions were continued until 1–2 consecutive additions failed to increase the respiration rate. For platelets, 4–13 injections of 1 µL of a 20 mM FCCP stock were given, resulting in end concentrations of 40 µM to 130 µM FCCP. For PBMC, about 1–4 injections of 0.5 µL of a 1 mM FCCP were given, resulting in end concentrations of 0.25 µM to 1 µM FCCP. The specific complex I inhibitor rotenone (1 mM stock; 1 µL) and, finally, the complex III inhibitor antimycin A (5 mM stock; 1 µL) were added for nonmitochondrial respiration, which is independent of the electron transfer chain activity. This residual oxygen consumption (ROX) that is not affected by these inhibitors is attributable to other cellular oxygen-consuming processes than the mitochondrial respiratory chain. Oxygen flux was quantified using DatLab software (version 5, OROBOROS Instruments, Innsbruck, Austria) and ROX was subtracted from ROUTINE, LEAK, and MAX OCRs for the evaluation of oxygen consumption specifically attributable to mitochondrial respiration. When after subtraction values were below zero, the values were set to zero. The final platelet concentration in the chamber was measured with an automated hematology analyzer (XN-10, Sysmex^®^, Kobe, Japan). The final PBMC concentration was measured using the Beckman Coulter DxH500. The OCRs were corrected for cell concentration. The LEAK/ET coupling-control ratio (L/E ratio) was calculated by dividing the corrected (ROX, cell concentration) LEAK by the corrected (ROX, cell concentration) MAX respiration rate. The L/E ratio is a measure for which the fraction of electron transfer system (ETS) capacity is related to non-phosphorylating respiration [36].

### 2.6. mtDNA Isolation from Plasma

The method to determine mtDNA levels was modified from Nakahira 2013 [16]. The DNA in the collected plasma was isolated using Qiagen DNeasy Blood & Tissue (#69504, Qiagen, Hilden, Germany). First, 125 µL of plasma was diluted with 125 µL PBS and mixed using a vortex. As we were interested in the free circulating mtDNA, we removed mtDNA-containing particles. For this purpose, we filtered 200 µL of the diluted plasma with a SpinX filter 0.22 µm (16,000× *g* 2 min) (#8160, Corning Costar, Salt Lake City, UT, USA). The filtrate was used for DNA isolation.

The remainder of the diluted plasma was used unfiltered to examine the difference between filtered and unfiltered. A total of 40 μL of the remaining diluted plasma was mixed with 160 μL PBS to obtain a final volume of 200 μL and was used directly for DNA isolation without filtering. 

For DNA isolation, 20 µL Proteinase K and 200 µL AL buffer (from DNA isolation kit) were added to the 200 µL filtrate. The samples were mixed and incubated at 56 °C for 15 min. After incubation, 200 µL of absolute ethanol was added and mixed using vortex. The samples were then transferred to a DNeasy isolation column from the kit, and the kit protocol was followed. In the final step, the DNA was eluted in 200 µL of eluent (AE buffer).

### 2.7. Quantative Polymerase Chain Reaction (qPCR)

To analyze the levels of mtDNA, the following primers were used: human NADH Dehydrogenase 1 (mtND1), forward primer: 5′-ATACCCATGGCCAACCTCCT-3′ and reverse primer: 5′-GGGCCTTTGCGTAGTTGTAT-3′. As a control for nuclear DNA, the following primers were used: human β-globin, forward primer: 5′-GTGCATCTGACTCCTGAGGAGA-3′ and reverse primer: 5′-CCTTGATACCAACCTGCCCAG-3′. A final primer concentration of 400 nM was used. 

To quantify the levels of mtDNA (mtND1) and nuclear DNA (β-globin) a gBlock gene fragment (synthetic dsDNA fragment, made by IDT Integrated DNA technologies, Leuven, Belgium) was used as a positive control. The gBlock gene fragments were provided dry and had to be resuspended in IDTE before first use, as described in the company’s instructions sheet. The concentration was checked with Nanodrop. The measured concentration was used for further calculations.

To convert the DNA concentration to a concentration in copy number/µL, the following formula was used:

C × M × (1 × 10^−15^ mol/fmol) × Avogrado number = copy number/µL

where C = current concentration in ng/µL (mtND1 = 19.3 ng/µL, β-globin = 14.7 ng/µL), M = molecular weight in fmol/ng (5.22 for mtND1 and 7.14 for β-globin according to the datasheet delivered with the gBlock), and Avogrado number = 6.022 × 10^23^.

The calculated copy number/µL in the stock solution for mtND1 and β-globin is 6.07 × 1010 copies/µL and 6.32 × 1010 copies/µL, respectively. A 10-fold dilution series from 6.07 × 106 copies to 6.07 × 102 copies (mtND1) and 6.32 × 106 copies to 6.32 × 102 copies (β-globin) was used as a standard.

The qPCR analysis was conducted using SensiMix SYBR & Fluorescein kit (#QT615–05, Bioline, Meridian Bioscience, Memphis, Tennessee) in combination with the Bio-rad CFX96 real-time system (Bio-Rad Laboratories, Singapore). The qPCR program used: 2 min at 50 °C and 10 min at 95 °C, then 40 cycles of 15 sec at 95 °C and 1 min at 58 °C. At the end, a melting curve analysis was performed to check amplification specificity. 

The data were analyzed using the qPCR software (Bio-Rad CFX manager 3.1, Bio-Rad Laboratories, Hercules, CA, USA), followed by further analysis in Excel and SPSS. Graphs were made with GraphPad version 9.

All samples and standards were measured in triplicates and a “no template control” (negative control) was included. The DNA samples were used undiluted. For converting the copies/µL DNA sample to copies/µL plasma, the following formula was used:

c = Q × (Vdna/Vpcr) × (1/Vext)

where c = copies/µL plasma, Q = copies calculated by qPCR software, Vdna = volume of extracted DNA (final step DNA isolation = 200 µL), Vpcr = volume of DNA used for qPCR (10 µL), and Vext = volume of plasma used for DNA isolation (filtered sample: Vext = 100 µL plasma/unfiltered sample: Vext = 20 µL plasma).

### 2.8. Data and Statistical Analysis

The sample size was based on earlier results in healthy volunteers and sepsis patients [27,37]. The calculation was performed with the program G*Power 3.1.9.7 [38]. Using the Wilcoxon–Mann–Whitney test, and assuming an effect size of 1.17, a sample size of 14 per group was calculated (α: 0.05, β: 0.80). To account for missing values, 16 COVID-19 patients and 16 healthy volunteers were included. 

To be able to obtain an adequate mitoPO_2_ and mitoVO_2_ measurement, enough protoporphyrin IX needs to be synthesized after application of the ALA plaster. If during a measurement the signal quality, as displayed by the COMET monitor, did not reach 20% for consecutive measurements, this was considered to be a measurement failure. The corresponding participant was excluded and replaced. 

Statistical analysis was performed using IBM Statistics SPSS 26 (SPSS Inc., Chicago, IL, USA). Graphpad Prism 9 (GraphPad Software, La Jolla, CA, USA) was used to make the figures. Demographic parameters were presented using descriptive statistics. Continuous variables are described as median, Q1, and Q3. Distribution of the data was visualized graphically using Q-Q plots and histograms. For the baseline characteristics between the COVID-19, healthy controls, and general anesthesia group, continuous data were tested using one-way ANOVA or the nonparametric equivalent. Categorical data were compared using a Pearson Chi-Square test. For comparison of continuous data between the COVID-19 groups and the healthy controls, the Mann–Whitney U test was used.

The cases and controls were only matched on age and gender and, therefore, nonpaired tests were chosen to compare COVID-19 patients to healthy controls. The Mann–Whitney U test was used to compare mitoPO_2_, mitoVO_2_, mitochondrial respiration in platelets and PBMCs, and mitochondrial DNA between COVID-19 patients and healthy controls. The Wilcoxon-signed rank test was used to compare the same parameters between the first and second time point. Depending on the variables, either Pearson correlation tests or Spearman correlation tests were used to assess correlations between mitoPO_2_, mitoVO_2_, mitochondrial respiration in platelets and PBMCs, mitochondrial DNA, and the SOFA and APACHE II scores. Outcomes were considered significant if *p* < 0.05. 

## 3. Results

### 3.1. Descriptive Statistics

**Figure 1:**
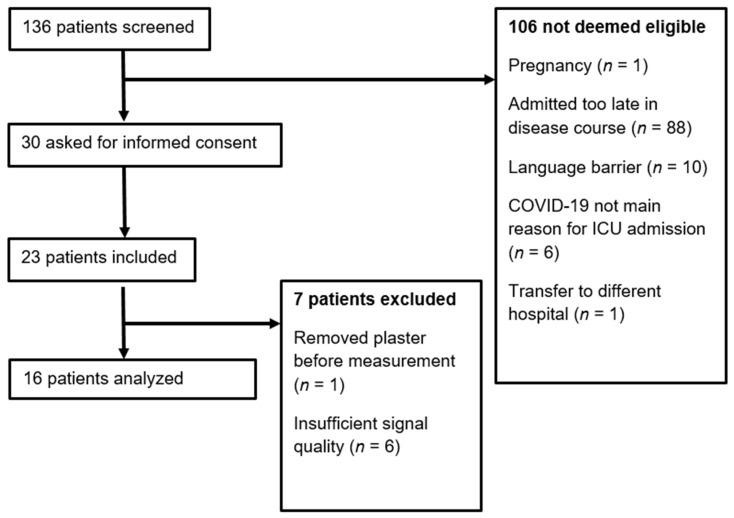
Flowchart of the inclusion process.

Of the 136 screened patients, a total of 16 patients were included in the study, as shown in Figure 1. Baseline patient characteristics are illustrated in Table 1. Out of the 16 included patients, 3 (19%) patients died during the study period on the ICU. At T1, data on mitochondrial respiration in PBMCs and mtDNA levels are missing for one patient. During T2, 14 out of the original 16 patients were measured with the COMET, as one patient was discharged and another patient refused the second measurement. Furthermore, at T2, both PBMC and PLT respiration data are missing for one patient. 

The time between intubation and measurements at T1 ranged from 1 to 3 days, with six (38%) of the patients being measured on day 2. A total of 12 (75%) of the included patients were male and median BMI was 31.5 [IQR; 27.5–34.5]. A total of 10 (63%) had a moderate ARDS score and a median SOFA score of 4.5 [IQR; 3–8] at admission to the local ICU, and a median APACHE II score of 22 [IQR; 18–24] during T1. In 13 (81%) patients, the COVID-19 lung phenotype was characterized as “ground glass opacities, without consolidations and pulmonary embolisms”. Furthermore, 11 (69%) patients followed the prone position ventilation regime, as per local protocol.

### 3.2. MitoPO_2_ Measurements

**Figure 2:**
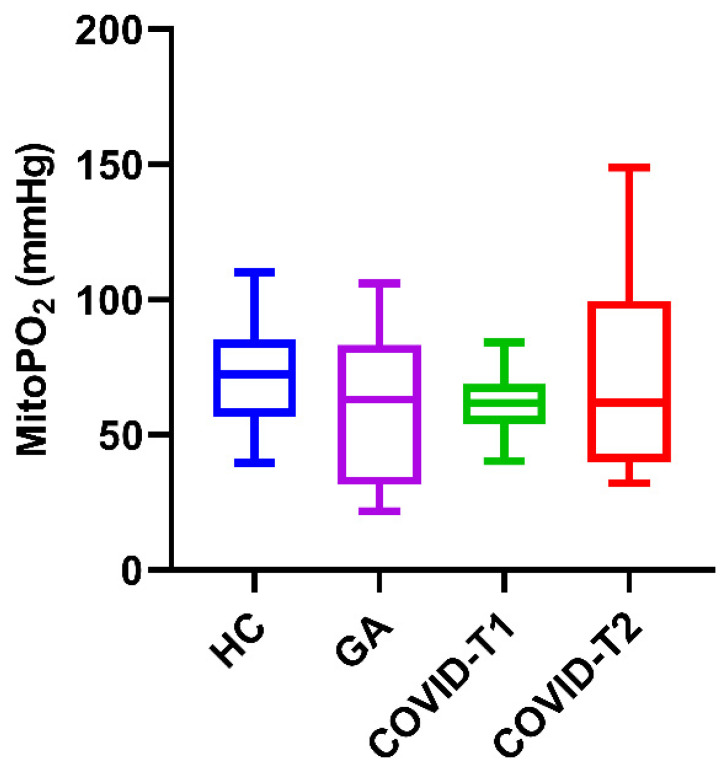
MitoPO_2_ (mmHg) in HC, COVID-19 patients on T1 and T2, and general anesthesia group. HC = healthy controls, T1 = time point 1, T2 = time point 2, and GA = general anesthesia. Values are displayed as median with interquartile range (box) and minimum and maximum (whiskers).

The median mitoPO_2_ of COVID-19 patients on T1 was 62 mmHg [IQR; 54–69]; this is identical to T2, with a mitoPO_2_ of 62 mmHg [IQR; 40–100] with a differing IQR. The median mitoPO_2_ in the healthy control group was 72 mmHg [IQR; 57–85]. Although the median mitoPO_2_ was higher in the healthy control group, there was no significant difference when comparing it against T1 (*p* = 0.122) or T2 (*p* = 0.480) of the COVID-19 cohort. The median mitoPO_2_ in the general anesthesia group was 63 mmHg [IQR; 34 79]; this did not differ significantly from the healthy controls and both COVID-19 time points. These results are visualized in the boxplot in Figure 2. Furthermore, there were no statistically significant differences between the mitoPO_2_ of the survival and mortality groups at COVID-T1 or COVID-T2.

There was no correlation found between the mitoPO_2_ measurements of the COVID-19 cohort and the APACHE II, ARDS category, and SOFA score. Similarly, there was no correlation between mitoPO_2_ and the filtered mtDNA.

### 3.3. MitoVO_2_ Measurements

**Figure 3:**
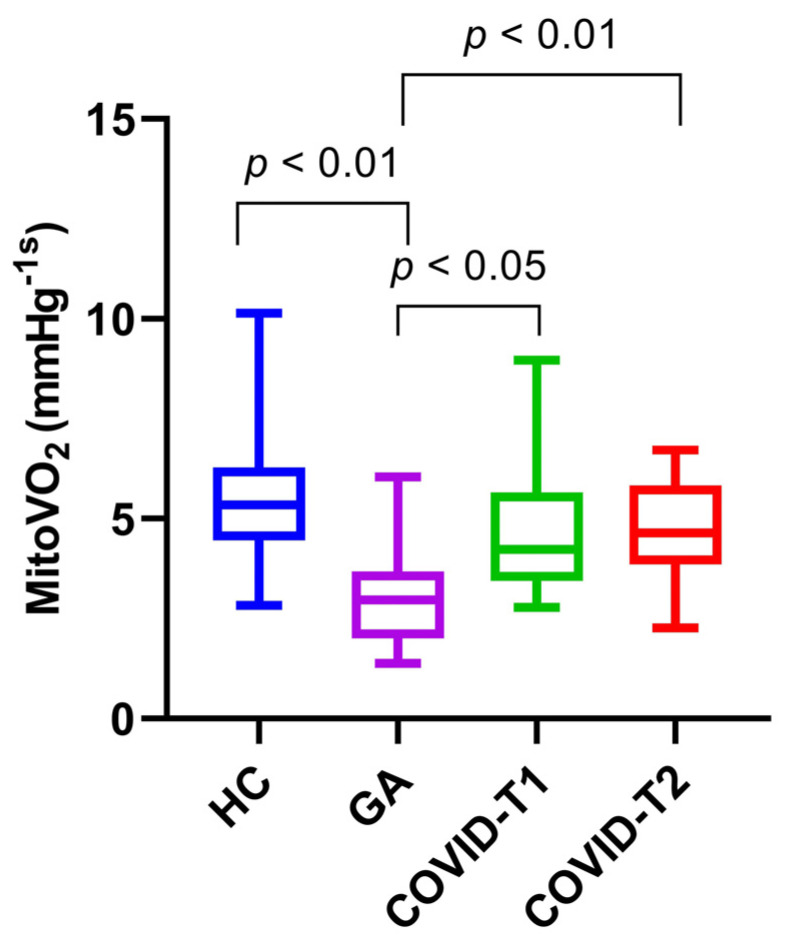
MitoVO_2_ (mmHg/s) in HC, COVID-19 patients on T1 and T2, and the general anesthesia group. HC = healthy controls, T1 = time point 1, T2 = time point 2, and GA = general anesthesia. Values are displayed as median with interquartile range (box) and minimum and maximum (whiskers).

The median mitoVO_2_ of COVID-19 patients on T1 was 4.6 mmHg s-1 [IQR; 3.6–6.0]; this is nearly identical to T2, with a mitoVO_2_ of 4.6 mmHg s-1 [IQR; 3.9–5.8] with a differing IQR. The median mitoVO_2_ in the healthy control group was 5.3 mmHg s-1 [IQR; 4.5–6.3]. The median mitoVO_2_ was higher in the healthy control group than in both COVID-19 time points, yet there was no significant difference when comparing it against T1 (*p* = 0.097) or T2 (*p* = 0.318) of the COVID-19 cohort. However, the mitoVO_2_ of the general anesthesia group was 3.0 mmHg s-1 [IQR; 2.2–3.4], which was significantly lower than the median mitoVO_2_ of COVID T1 (*p* = 0.017), COVID T2 (*p* = 0.004), and healthy controls (*p* = 0.001). These results are portrayed in the boxplot in Figure 3. Moreover, there were also no statistically significant differences between the mitoVO_2_ of the survival and mortality groups at COVID-T1 or COVID-T2. Lastly, there were no correlations between the mitoVO_2_ measurements of the COVID-19 cohort and the APACHE II score, ARDS category, and SOFA score. 

### 3.4. Whole Blood Cell Count

**Figure 4:**
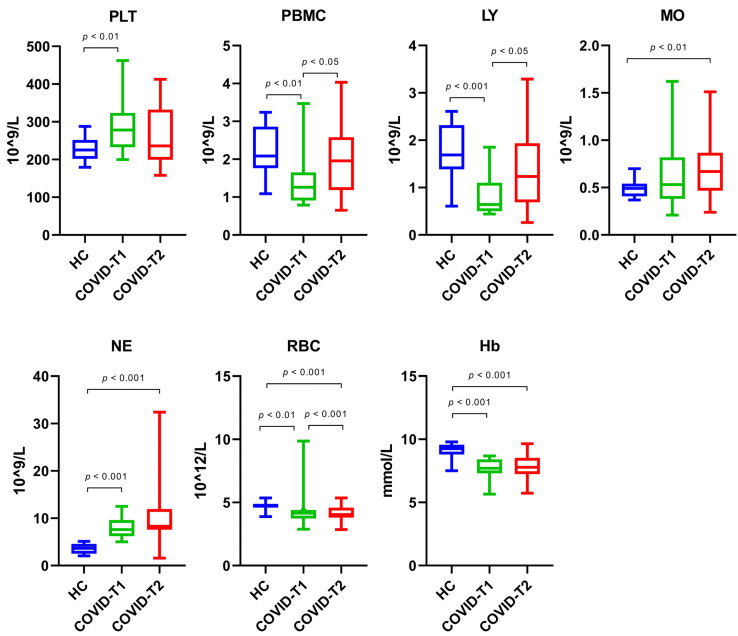
Whole blood cell counts for platelets (PLT), PBMC, lymphocytes (LY), monocytes (MO), neutrophils (NE), red blood cells (RBC), and Hb levels for the healthy control (HC), COVID-T1, and COVID-T2 groups.

Whole blood cell counts are displayed in Figure 4. Whole blood platelet counts were 23.6% higher in the COVID-19 patients at T1 when compared to the healthy controls. At T2, the platelet count had normalized. PBMCs were 39.6% lower at T1 in the COVID-19 patients when compared to the healthy controls. Similar to the platelet count, PBMC had normalized at T2 in the COVID-19 patients. The majority of the decrease in PBMCs can be attributed to a decrease in lymphocytes (62.1%). No significant change was observed in monocytes at T1 in the COVID-19 patients. Lymphocytes were increased by 93% at T2 compared to T1 in the COVID-19 patients, while monocytes were increased by 26.4% between the two time points. In comparison to the healthy controls, neutrophil numbers were increased by two-fold at T1 (108.5%) and T2 (127.8%). Both the amount of RBC (12.4% (T1) and 14.5% (T2)) and the hemoglobin levels (16.6% (T1) and 15.9% (T2)) were lower than the levels measured in the healthy controls. 

### 3.5. Isolated Blood Cell Count

The purity from the cells isolated from the healthy control group was high, with a mean of 94.9% of the cells being PBMC (lymphocytes + monocytes) and 4.9% neutrophils. However, isolation of PBMC from both COVID-19 groups yielded lower numbers, with 62.8% and 80.1% PBMC at T1 and T2 (36.4% and 19.2% neutrophils). 

### 3.6. Platelet Oxygen Consumption

**Figure 5:**
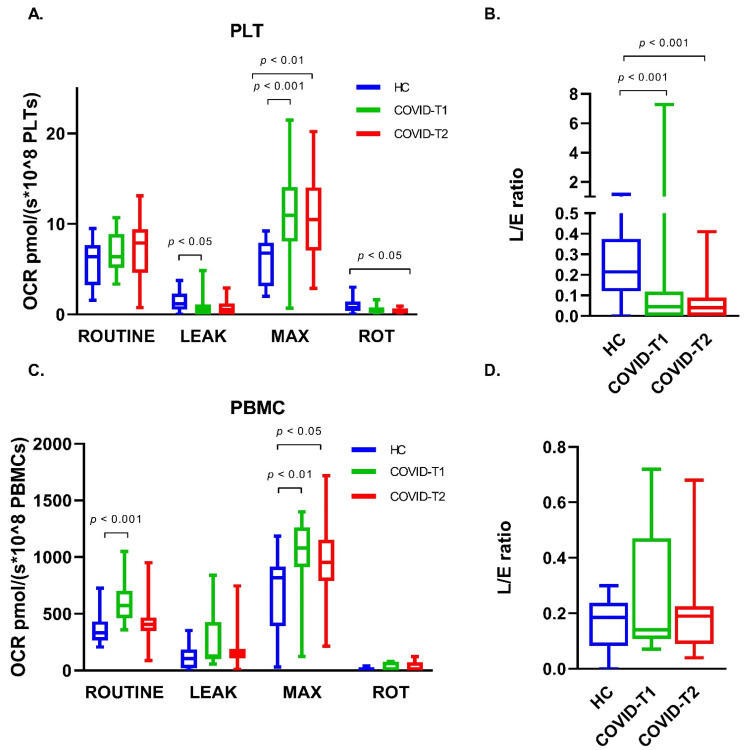
Oxygen consumption rate (OCR) for ROUTINE, LEAK, maximal respiration (MAX), and respiration after inhibition of complex I by rotenone (ROT) in (**A**) platelets and (**C**) PBMC. LEAK/ET coupling control ratio (L/E Ratio) in (**B**) platelets and (**D**) PBMC for the healthy control (HC), COVID-T1, and COVID-T2 groups. Values displayed as median ± min/max values.

Platelet ROUTINE OCR in both COVID-19 groups was not different from the healthy controls (*p* = 0.432, *n* = 16 (T1) and *p* = 0.379, *n* = 15 (T2)) (Figure 5A). A small decrease in leak OCR was observed for both COVID-19 groups compared to the healthy controls (*p* = 0.052, *n* = 16 (T1) and *p* = 0.036, *n* = 15 (T2)). While platelets from healthy controls showed no increase in OCR after stimulation with FCCP, maximal OCR was increased by 59.9% and 50.9% in the COVID-T1 and T2 group, respectively. OCR after inhibition of complex I with rotenone (ROT) was reduced to a very minimal rate. The LEAK/ET coupling control ratio (L/E ratio) decreased, with 79.9% in the COVID-T1 group and with 82.7% in the COVID-T2 group compared to the healthy controls (Figure 5B).

### 3.7. PBMC Oxygen Consumption

PBMC ROUTINE OCR in the COVID-T1 group was increased, with 77.5% compared to healthy controls, while OCR levels in the COVID-T2 group were similar to healthy controls ROUTINE OCR levels (Figure 5C). No significant differences in leak OCR were observed between the three groups. Maximal stimulation of OCR with FCCP resulted in a 70.5% increase in OCR compared to ROUTINE for the healthy control group and a similar 67.2% increase for the COVID-T1 group, while maximal OCR in the COVID-T2 group increased, with 95.6%. OCR after inhibition of complex I with rotenone (ROT) was completely abolished. No differences in L/E ratio between the healthy controls and both the COVID-19 groups were found (Figure 5D).

### 3.8. In Vivo and Ex Vivo Measurement Correlation Analysis

No correlation could be found between the mitoVO_2_ and basal aerobic respiration in platelets, as analyzed using the Oroboros technique, at T1 (rs = 0.221, *p* = 0.421, *n* = 16) nor at T2 (rs = 0.280, *p* = 0.354, n = 13). Neither could this be found for the PBMCs at T1 (rs = −0.108, *p* = 0.714, *n* = 14), nor at T2 (rs = −0.468, *p* = 0.091, *n* = 14). Likewise, there was no correlation between the filtered mtDNA and the mitoVO_2_ at T1 (rs = −0.268, *p* = 0.334, *n* = 15) nor at T2 (rs = −0.002, *p* = 0.994, *n* = 14). 

### 3.9. Nuclear DNA and mtDNA Amounts in Plasma

**Figure 6:**
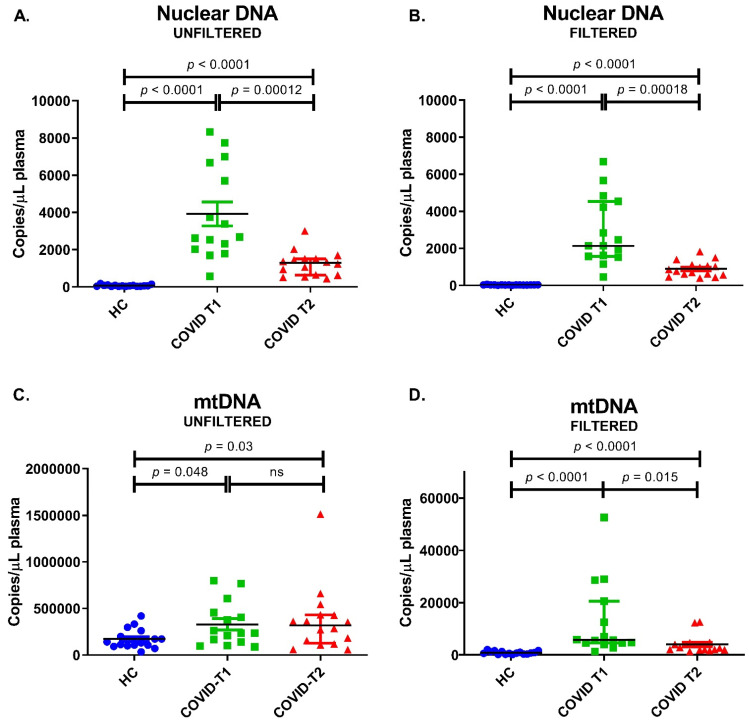
Plasma amount of nuclear DNA (β-globin) and mitochondrial DNA (mtDNA) in HC = healthy controls, COVID-T1, and COVID-T2 groups. (**A**) Unfiltered nuclear DNA amount. (**B**) Filtered nuclear DNA amount. (**C**) Unfiltered mtDNA. (**D**) Filtered mtDNA. Values are displayed as median ± IQR.

Low levels of nuclear DNA (β-globin) were present in healthy control samples (median 55 copies/µL plasma). These were 48-fold higher in COVID-T1 samples and 23-fold higher in COVID-T2 samples when compared to healthy control samples (Figure 6A). Filtration reduced nuclear DNA levels by 50, 20, and 37% for the healthy controls, COVID-T1, and COVID-T2 samples, respectively (Figure 6B). 

mtDNA was detectable in unfiltered samples of healthy controls (median 135,000 copies/μL plasma) and was two-fold higher in both COVID-19 groups (Figure 6C). As described in the methods section, in order to measure free-floating mtDNA and not a combination of free mtDNA and mtDNA-containing particles, filtration was performed. Filtration reduced total mtDNA levels by 99.5, 97.6, and 99.1% for the healthy controls, COVID-T1, and COVID-T2 samples (Figure 6D). Free mtDNA levels in the COVID T1 samples were eight-fold higher compared to healthy controls, while mtDNA in the COVID-T2 samples was two-fold lower compared to COVID-T1 mtDNA levels (Figure 6D). An overview of the measured copies/µL plasma of both β globin and mtDNA can be found in Table 2. 

## 4. Discussion

This study is the first to examine in vivo mitoPO_2_ and mitoVO_2_ in critically ill SARS-CoV-2 sepsis patients admitted to the ICU. The mitoPO_2_ and mitoVO_2_ were measured within 72 h of admission and 7 days postadmission to the ICU of a tertiary university hospital. The measurements were conducted through the use of a COMET*^®^* device based on the PpIX-TLST to monitor oxygen delivery and consumption on a cellular level, thereby providing insight into in vivo mitochondrial function [24]. Furthermore, this study also utilizes ex vivo measurements to examine mitochondrial function, namely PBMC and platelet oxygen consumption, as well as analyzing the free circulating mtDNA, which is a potential biomarker for sepsis.

### 4.1. MitoPO_2_ and mitoVO_2_

The in vivo COMET^®^ measurements conducted in this study found no significant differences between the COVID-19 patients and the age-matched control group. The mitoPO_2_ remained almost identical in the SARS-CoV-2 patient group at time point 1 and 2 and was not significantly lower than that of the healthy controls. The mitoVO_2_ was also identical at time point 1 and time point 2 and was not substantially lower than that of the healthy controls. Our results are in contrast with a previous study. In 2020, Neu et al. published a pilot study examining the feasibility of mitoVO_2_ measurements in ICU critically ill patients [37]. They found that the median mitoVO_2_ for this critically ill group was 3.3 mmHg s-1, as opposed to 4.6 mmHg s-1 in both our COVID-19 time points. The patients included in Neu’s study did not have COVID-19, as their data collection was completed before the pandemic. This may suggest that severe COVID-19 results in increased mitochondrial respiration compared to other critically ill patients. 

In line with this reasoning, mitoVO_2_ was significantly lower in the general anesthesia group when compared to the COVID-19 cohort and the healthy controls. This highlights the effects that anesthetics have on the mitochondria, as almost every general anesthetic depresses mitochondrial function, even at concentrations commonly used in the operating room [39,40,41,42]. This suggests that the measured severe COVID-19 patients are actually in a relative hypermetabolic state, as they have a significantly higher mitoVO_2_ than the general anesthesia group and they maintained a similar mitochondrial function to that of the healthy controls in this study who did not receive any anesthetics.

Another technique by which the metabolic rate can be examined is through indirect calorimetric testing. Niederer et al. examined the resting energy expenditure using this technique in severe COVID-19 patients admitted to the ICU [43]. They found that severe COVID-19 patients exhibit a continuous hypermetabolic state for up to 7 weeks postintubation. The authors went on to conclude that this is unique to COVID-19 patients, as sepsis patients usually exhibit a short transitory hypermetabolic phase, which peaks within days and is followed by a hypometabolic state [43,44]. However, to fully compare the data in our study with those of Niederer et al., a longer period of measurement is required. 

These results suggest that there is an elevated mitochondrial respiration, as depicted by the mitoVO_2_ in critically ill COVID-19 patients, especially when considering the depressive effects of anesthetics on mitochondrial function, as illustrated by the general anesthesia group. 

### 4.2. Mitochondrial DNA

Free circulating mtDNA is a potential biomarker for sepsis, as it is associated with disease severity and mortality [18,45]. In line with the results of previous studies in critically ill (sepsis) patients, mtDNA levels of SARS-CoV-2 patients were elevated in comparison to healthy controls [17,46]. In our results, mtDNA was significantly higher in the COVID-19 cohorts compared to the healthy controls. However, it was not associated with disease severity. Currently, two published clinical studies have analyzed mtDNA in SARS-CoV-2 patients. Valdés-Aguayo et al. measured mtDNA in whole blood from patients with SARS-CoV-2 disease and demonstrated that patients with severe SARS-CoV-2 disease had lower levels of mtDNA compared to patients with mild SARS-CoV-2 disease [47]. The comparison of our study with that of Valdés-Aguayo is not possible, as they measured the mtDNA in whole blood instead of blood plasma. 

Scozzi et al. reported on the predicting factor of cell-free plasma mtDNA levels for morbidity and mortality of COVID-19. Their results demonstrated higher levels of mtDNA at hospital admission in patients who were admitted to the ICU, intubated, and died during their illness trajectory compared to patients who did not [48]. However, a comparison of their findings to ours is not completely feasible, as the timing of patient inclusion was different. Disease progression of severe COVID-19 can be divided into four phases: early infection, host immune response, hyperinflammatory phase, and multiorgan dysfunction [49]. It can be deduced that, in regards to the proposed phases of disease, the two studies have measured at different time points. Additionally, due to the fact that Scozzi et al. measured at hospital admission, they facilitated the inclusion of a diverse patient population, enabling them to be able to compare mtDNA levels between different disease severity categories. As previously mentioned, the patients included in our study were at a further stage of disease progression as they were admitted to the ICU.

Moreover, Chiu et al. concluded that mtDNA in plasma can either be particle-associated or free mtDNA and, as a result, the amount of measured mtDNA is highly dependent on preparation protocols, specifically on filtration steps [50]. Therefore, the comparison of mtDNA levels between studies is only realistic if mtDNA is not only measured in the same compartment, but also using the same preparation protocol.

### 4.3. PBMCs 

Similar to the in vivo result, no change in basal oxygen consumption was observed between isolated platelets from healthy controls and from the SARS-CoV-2 patient groups at time point 1 and 2. In contrast, the basal mitochondrial respiration in isolated PBMC was increased in the SARS-CoV-2 patients at time point 1 compared to control but had normalized at time point 2. 

Preceding research in SARS-CoV-2 patients using high-resolution respirometry has been performed by Gibellini et al. who found reduced basal and maximal respiration, reduced proton leak, and reduced spare capacity in monocytes, assigning this to dysfunctional and metabolically impaired mitochondria [14]. Similarly, Ajaz et al. described decreased basal and maximal respiration in PBMCs of SARS-CoV-2 patients admitted to the ICU compared to patients with a chest infection and healthy controls [15]. Although our results are not in line with theirs, variating results of mitochondrial respiration and function in PBMCs and platelets have been described in sepsis research previously [29,51,52,53,54]. The differing results could imply that, during different stages of disease, mitochondrial respiration is either increased or decreased. However, as Jeger et al. suggest in their review, these outcomes could also be attributed to high (biological) variability of mitochondrial respiration or different experimental conditions [54]. Large standardized trials which monitor mitochondrial respiration for several days in both PBMCs and platelets are needed to elucidate mitochondrial function in SARS-CoV-2 and sepsis.

### 4.4. Limitations

The in vivo mitochondrial function measurements were only conducted on critically ill SARS-CoV-2 patients admitted to the ICU and healthy controls. This limits the comparison, as there was no critically ill patient group that we could contrast to the SARS-CoV-2 critically ill patients. In order to overcome this limitation for at least the effects of anesthetics on the mitoVO_2_, a general anesthesia group was added to this manuscript. We realize that this is a suboptimal control group, mainly due to the fact that these patients have a different underlying pathology than the COVID-19 patients. However, they do tend to have similar comorbidities, as shown in Table 1. Moreover, as this general anesthesia cohort originated from another study protocol, the ex vivo mitochondrial biomarkers and mitochondrial function analyses could not be conducted. Furthermore, this study examined the mitochondrial oxygenation and function within 72 h of tertiary ICU admission, potentially missing the early hyper inflammatory phase of SARS-CoV-2, as some patients were already admitted and intubated on the ICU in a smaller medical center before admission to the Erasmus Medical Center [55].

## 5. Conclusions

The present results suggest an elevated oxygen metabolism in COVID-19 patients and concurrent mitochondrial damage compared to healthy controls. Further research should clarify the effect of the different phases of COVID-19 disease on mitochondrial function and the differences in mild, moderate, and severe disease.

## Figures and Tables

**Table 1:** Baseline characteristics. Values are displayed as n (%) or median [IQR].

	COVID-19 Patients	Healthy Control	General Anesthesia	*p*-Value
Gender (M/F)	12/4	12/4	7/2	*p* = 0.913
Age	59 (52–65)	57 (53–63)	66 (58–70)	*p* = 0.397
Body Mass Index	31.45 (27.54–34.47)	24.91 (22.93–27.34)	26.49 (24.22–28.49)	*p* = 0.002
Comorbidity (n,%)				
• Hypertension	8 (50%)	0	1 (11%)	*p* = 0.002
• Coronary artery disease	1 (6.25%)	0	5 (55%)	*p* < 0.001
• Diabetes Mellitus	3 (18.6%)	0	1 (11%)	*p* = 0.200
• Obesity	10 (62.5%)	1 (6.25%)	2 (22%)	*p* = 0.004
• Morbid Obesity	2 (12.5%)	0	0	*p* = 0.193
• Astma or COPD	2 (12.5%)	1 (6.25%)	1 (11%)	*p* = 0.827
• Kidney disease	1 (6.25%)	0	0	*p* = 0.449
Time between intubation and first measurement				
• 1 days	5 (31%)	n.a.	n.a.	n.a.
• 2 days	6 (38%)	n.a.	n.a.	n.a.
• 3 days	4 (25%)	n.a.	n.a.	n.a.
SOFA score on ICU admission	4.50 (3.00–8.00)	n.a.	n.a.	n.a.
Apache II score				
• COVID-T1	22.00 (18.25–23.75)	n.a.	n.a.	n.a.
• COVID-T2	9.50 (7.25–19.75)	n.a	n.a	n.a.
ARDS score (n,%)				
• Mild	3 (19%)	n.a.	n.a.	n.a.
• Moderate	10 (62.5%)	n.a.	n.a.	n.a.
• Severe	2 (12.5%)	n.a.	n.a.	n.a.
Prone position (n,%)	11 (69%)	n.a.	n.a.	n.a
CT-scan phenotype				
• Ground glass opacities, without consolidations and pulmonary embolisms	13 (81.25%)	n.a.	n.a.	n.a.
• Pulmonary embolism in combination with ground glass opacities	2 (12.5%)	n.a.	n.a.	n.a.
• Consolidations accompanied by pulmonary fibrosis and ground glass opacities consistent with ARDS	1 (6.25%)	n.a.	n.a.	n.a.
Whole blood cell count				
• Platelets	278 (233–324)	225 (202–252)	n.a.	*p* = 0.003
• Peripheral blood mononuclear cells	1.26 (0.91–1.65)	2.09 (1.76–2.86)	n.a.	*p* = 0.002
• Lymphocytes	0.64 (0.50–1.10)	1.69 (1.39–2.32)	n.a.	*p* < 0.001
• Monocytes	0.53 (0.38–0.82)	0.49 (0.41–0.54)	n.a.	*p* = 0.514
• Neutrophils	7.62 (6.20–9.60)	3.66 (2.52–4.59)		*p* < 0.001
• Red blood cell	4.15 (3.73–4.41)	4.74 (4.60–4.90)	n.a.	*p* = 0.003
• Hemoglobin	7.71 (7.31–8.41)	9.25 (8.79–9.58)	n.a.	*p* < 0.001

n.a. = not applicable.

**Table 2:** Plasma amount of nuclear DNA (β-globin) and mitochondrial DNA (mtDNA) in HC = healthy controls, COVID-T1, and COVID-T2 groups before and after filtration. Filtered mtND1. (*n* = 15–16). Values are displayed as median (IQR).

		Unfiltered Plasma	Filtered Plasma	
		mtDNA-Containing Particles + Free Circulating mtDNA	Free Circulating mtDNA	
Nuclear DNA (β-globin)	HC (*n* = 16)	55 (33–87)	28 (22–36)	copy/µL plasma
	COVID-T1 (*n* = 15)	2680 (2030–6670)	2140 (1572–4540)	
	COVID-T2 (*n* = 16)	1290 (637–1510)	818 (576–1101)	
mtDNA (mtND1)	HC (*n* = 16)	135,000 (101,350–245,500)	729 (353–1282)	
	COVID-T1 (*n* = 15)	236,000 (141,000–455,000)	5720 (4600–20,600)	
	COVID-T2 (*n* = 16)	319,500 (127,000–430,000)	2750 (1980–4490)	

No statistically significant correlations were found between filtered mtDNA and the APACHE II score (rs = −0.193, *p* = 0.336, *n* = 27), SOFA score (rs = −0.069, *p* = 0.808, *n* = 15), or mortality (rs = −0.087, *p* = 0.643, *n* = 31) outcomes.

## Data Availability

Data is contained within the article.
